# Study protocol: a prospective single-center study for non-invasive biomonitoring of renal complications in cancer patients treated with immune checkpoint inhibitors

**DOI:** 10.3389/fimmu.2023.1140677

**Published:** 2023-04-26

**Authors:** Eva Baier, Peter Korsten, Arne Strauß, Kai-Martin Thoms, Tobias Overbeck, Philipp Ströbel, Björn Tampe

**Affiliations:** ^1^ Department of Nephrology and Rheumatology, University Medical Center Göttingen, Göttingen, Germany; ^2^ Department of Urology, University Medical Center Göttingen, Göttingen, Germany; ^3^ Department of Dermatology, University Medical Center Göttingen, Göttingen, Germany; ^4^ Department of Hematology and Medical Oncology, University Medical Center Göttingen, Göttingen, Germany; ^5^ Institute of Pathology, University Medical Center Göttingen, Göttingen, Germany

**Keywords:** PD-L1, AIN, irAE, ICI, urinary flow cytometry, TEC

## Abstract

**Background:**

The advent of immune checkpoint inhibitors (ICIs) has powerfully broadened the scope of treatment options for malignancies with an ongoing increase of indications, but immune-related adverse events (irAEs) represent a serious threat to treatment success. Agents directed against programmed cell death protein 1 (PD-1) or its ligand 1 (PD-L1) are known to cause renal complications with an incidence of 3%. In contrast, subclinical renal involvement is estimated to be much higher, up to 29%. We recently reported about urinary flow cytometry-based detection of urinary PD-L1-positive (PD-L1^+^) kidney cells correlating with tubular PD-L1-positivity that reflected susceptibility to develop ICI-related nephrotoxicity as an irAE attending ICI treatment. Therefore, we designed a study protocol to evaluate urinary detection of PD-L1^+^ kidney cells as a tool for non-invasive biomonitoring of renal complications in cancer patients treated with ICIs.

**Methods:**

A prospective, controlled, non-interventional, longitudinal, single-center observational study will be conducted at the Department of Nephrology and Rheumatology of the University Medical Center Göttingen, Germany. We intend to enroll approximately 200 patients treated with immunotherapy from the Departments of Urology, Dermatology, and Hematology and Medical Oncology of the University Medical Center Göttingen, Germany. First, we will assess clinical, laboratory, histopathological, and urinary parameters in addition to urinary cell collection. Then, we will perform a correlative analysis between urinary flow cytometry of different PD-L1^+^ cell of renal origin with the onset of ICI-related nephrotoxicity.

**Discussion:**

Because of growing ICI-treatment applicability with an expectable incidence of renal complications, providing cost-efficient and easily performable diagnostic tools for treatment-attendant and non-invasive biomonitoring becomes vital to improve both renal and overall survival rates in cancer patients receiving immunotherapy.

**Trial registration:**

https://www.drks.de, DRKS-ID DRKS00030999.

## Introduction

With the advent of immune-checkpoint inhibitors (ICIs), the scope of cancer-treatment options has seen a powerful increment, wherein reactivation of CD8-positive T-cell cytotoxicity constitutes its functional ground (1). Established neutralizing antibodies are directed against co-inhibitory auxiliary proteins expressed on tumor cells engaging in mechanisms of so-called immune evasion. Blockade of programmed cell-death protein 1 (PD-1) and its ligand 1 (PD-L1) is associated with remarkable therapy responses, especially for solid tumor entities featuring restricted therapeutic options. Still, immune-related adverse events (irAEs) pose a serious threat to treatment success, including maintenance of tumor remission (2–6). Of these irAEs, skin, gastrointestinal, hepatic, and endocrine adverse effects are the most common (7). Kidney involvement is known to occur with an incidence ranging from 2 to 3%; subclinical affection is estimated even higher, up to 29% (8–16). Glucocorticoid therapy and discontinuation of the causative agent are the only available measures with the side effect of delayed cancer treatment. Once affected, kidneys are more prone to a renal relapse after ICI re-exposition (9–11). Nephrotoxicity related to ICI therapy mainly consists of acute interstitial nephritis (AIN) and, to a lesser extent, glomerular nephropathies (8–19). This nephrotoxicity remains unclear and deserves to be characterized regarding clinical patterns and the underlying mechanisms.

We previously reported aberrant PD-L1 expression distinct to renal compartments in ICI-related nephrotoxicity, reflecting susceptibility to develop renal irAE (20, 21). PD-L1 positivity is different in intrarenal compartments and predominantly expressed in the tubuli, which correlates with elevated serum levels of C-reactive protein (CRP) as a non-specific marker of systemic inflammation (20). Moreover, PD-L1 expression was also observed in ICI-naïve renal pathologies implying PD-L1 upregulation as an indicator of ongoing kidney damage before this becomes clinically detectable by conventional methods (20). Interestingly, urinary flow cytometry-detected PD-L1-positive (PD-L1^+^) kidney cells correlated with intrarenal PD-L1 positivity (20). Therefore, we designed a study protocol to evaluate urinary detection of PD-L1^+^ kidney cells of different origins as a tool for non-invasive and therapy-attendant biomonitoring of renal complications in cancer patients receiving immunotherapy.

## Methods

### Study design and study population

Our observational single-center study is prospective, controlled, longitudinal, and non-interventional (trial registration: https://www.drks.de, DRKS-ID: DRKS00030999). It will be performed at the Department of Nephrology and Rheumatology at University Medical Center Göttingen (UMG), Germany. In addition, cancer patients receiving ICI treatment at the Departments of Dermatology, Urology, Hematology, and Medical Oncology of the UMG will be enrolled. The type and dosing schedule of the ICI therapy will be performed as indicated by the responsible medical specialist and carried out independently from the study investigations. A sample size of 200 patients was calculated based on the reported incidences of ICI-related nephrotoxicity.

### Patient enrollment and study conduction

**Figure 1:**
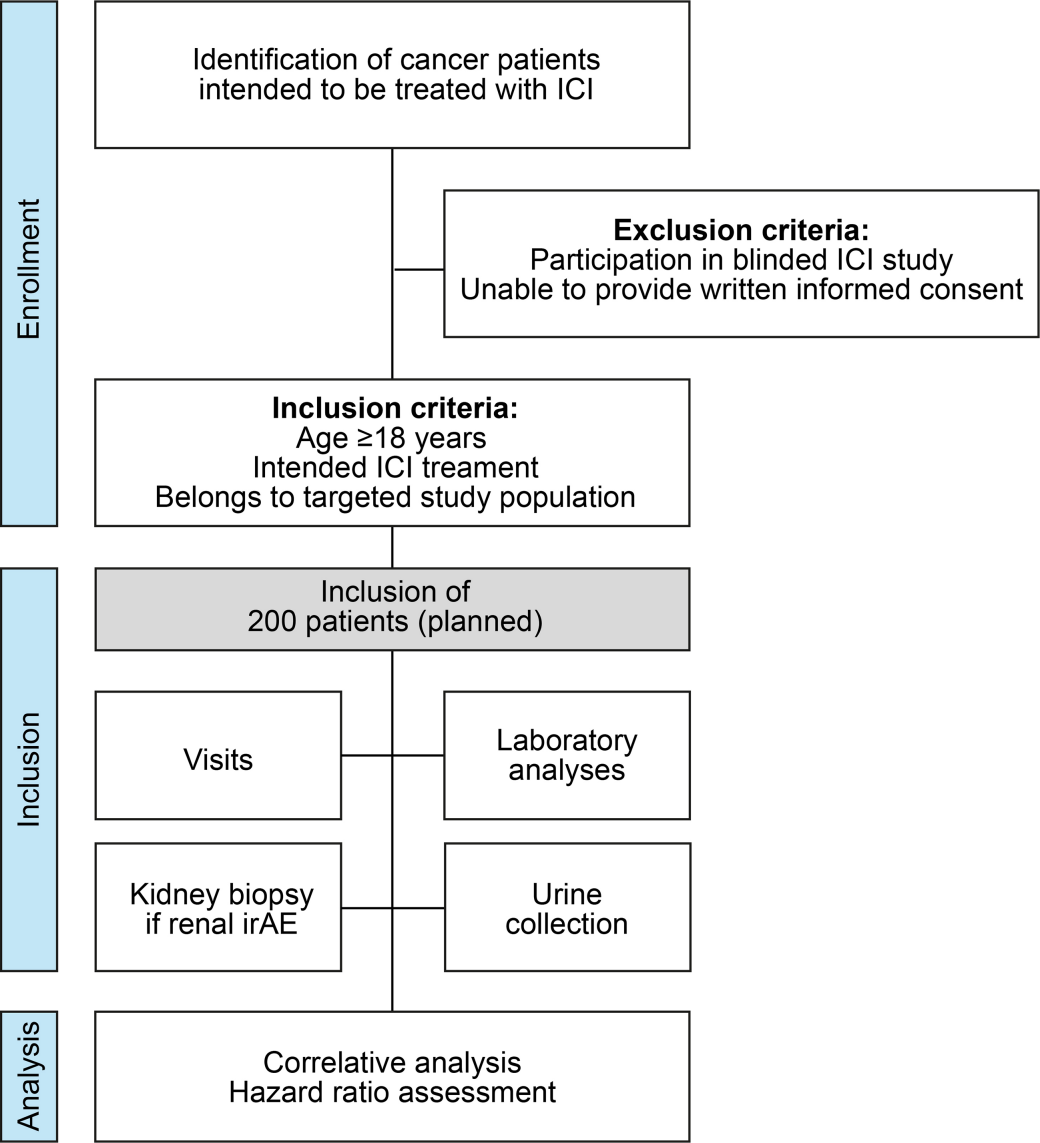
STROBE flow chart of the study protocol. ICI, immune checkpoint inhibitor; irAE, immune-related adverse event; STROBE, strengthening the reporting of observational studies in epidemiology.

Inclusion criteria are patients 18 years or older, written informed consent, and intended immunotherapy. In addition, patients enrolled in other ongoing trials of immunotherapies with a blinded study design are considered ineligible. Within the framework of routine medical care of patients in the respective departments, eligible patients are identified and included after documented written consent. **Figure 1** shows a STROBE flow chart of the study protocol featuring an overview of inclusion and exclusion criteria. After providing written consent, a baseline visit will be conducted, where baseline demographic data, medical history, medication, and clinical symptoms will be collected. Physical examination includes the assessment of height, weight, vital parameters, general condition, the status of the integument, neck with the thyroid gland, head, ears, eyes, throat, abdomen, limbs, joints, and auscultation of the heart and lungs. Laboratory data assessments include differential blood cell counts, serum levels of sodium, potassium, calcium, creatinine, blood urea nitrogen (BUN), uric acid, aspartate aminotransferase (AST), alanine aminotransferase (ALT), gamma-glutamyl transferase, bilirubin, lactate dehydrogenase (LDH), lipase, blood glucose, creatine kinase (CK), thyroid-stimulating hormone (TSH), T3, T4, and CRP. In addition, viral serology for the human immunodeficiency virus (HIV), hepatitis A, B, and C, cytomegalovirus (CMV), and the Epstein-Barr virus (EBV) will be tested at baseline. Moreover, autoantibody testing will be performed once at baseline and afterward as clinically indicated and includes antinuclear antibodies (ANA), anti-neutrophilic cytoplasmic antibodies (ANCA), extractable nuclear antigen (ENA), anti-double stranded desoxyribonucleic acid (anti-dsDNA), anti-cyclic citrullinated peptide (anti-CCP) and rheumatoid factor (RF). In addition, urinanalysis will be performed at each visit with urine dipstick and urine sediment, and an additional asservation of 100 mL of fresh urine samples will be performed. Follow-up visits are scheduled every 12 (–16) weeks; unscheduled visits will be performed if an irAE is suspected. The end-of-study visit will be conducted after 48-52 weeks. The schedule of visits and assessments is shown in **Table 1**.

**Table 1:** Schedule of patient visits and assessments.

Week/occasion	0	12 (+4)	48 (+4)	Routine visits during ICI application	Non-scheduled visits (occurrence of irAE)
Written informed consent	X				
In-/exclusion criteria	X				
Enrollment	X				
Medical history	X	X	X	X	X
Physical examination^1^	X	X	X	X	X
Vital parameters^2^	X	X	X	X	X
Weight and height^3^	X	X	X	X	X
Laboratory analyses^4^	X	X	X	X	X
Urinary analyses^5^	X	X	X	X	X
Viral serologies^6^	X				
Antibody screening^7^	X				X
Medication	X	X	X	X	X
Assessment of irAE^8^	X	X	X	X	X

^1^Includes general condition, status of the integument, neck with thyroid, head, ears, eyes, throat, abdomen, limbs, joints and auscultation of heart and lungs, orienting neurological examination.

^2^Blood pressure and heart rate after five minutes of rest.

^3^Weight in kilogram, height in centimeters.

^4^Including differential blood cell count, serum levels of sodium, potassium, calcium, creatinine, BUN, uric acid, AST, ALT, gGT, bilirubin, LDH, blood glucose, CK, TSH, T3, T4, CRP.

^5^Urinary dipstick, urinary sediment, 100 mL fresh urine sample.

^6^HIV, hepatitis A/B/C, CMV, EBV.

^7^RF, anti-CCP, ANA, ENA, ANCA, anti-dsDNA.

^8^Documented on a separate data sheet.

ALT, alanin aminotransferase; ANA, antinuclear antibodies; ANCA, anti-neutrophilic cytoplasmic antibodies; Anti-CCP, cyclic citrullinated peptide; Anti-dsDNA, anti-double stranded desoxyribonucleic acid; AST, aspartate aminotransferase; BUN, blood urea nitrogen; CK, creatine kinase; CMV, cytomegalovirus; CRP, C-reactive protein; EBV, Epstein-Barr virus; ENA, extractable nuclear antigen; gGT, gamma-glutamyl transferase; HIV, human immunodeficiency virus; ICI, immune checkpoint inhibitor; irAE, immune-related adverse event; LDH, lactate dehydrogenase; RF, rheumatoid factor; TSH, thyroid-stimulating hormone.

### Asservation of urine cells

Fresh urine samples will be collected (100 mL) and loaded into an automated cytospin machine (Shandon cytospin, Thermo Scientific, Pittsburgh, USA) following the manufacturer’s instructions and centrifuged at 1000 revolutions per minute (rpm) for 10 minutes. The cell pellet is resuspended in a mixture of phosphate-buffered saline (PBS) and bovine serum albumin (BSA). Cell viability and quantity are measured in a cell counter. Urine cells are deep frozen in Gibco™ CTS™ Synth-a-Freeze™ medium at -80°C. Cells can be further processed for urinary flow cytometry.

### Flow cytometry of urinary cells

Antibody staining of cells in suspension follows standard protocols. Thawed or freshly collected cells are centrifuged at 1000 rpm at 4°C for 8 minutes. The PD-L1^+^ cell line KARPAS 299 will be used as a positive control and unstained cells as negative controls for the analysis of urinary cells. Cell suspensions are washed and resuspended in PBS and stained using antibodies against PD-L1 (APC anti-human CD274, B7-H1, PD-L1, clone 29E.2A3, Biolegend, San Diego, USA) in combination with the podocyte marker podocalyxin (anti-TRA-1-81-PE, human, clone REA246, Miltenyi Biotec GmbH, Bergisch Gladbach, Germany). Identification of tubular epithelial cell (TEC) will be done by cytokeratin (anti-cytokeratin-FITC, human, clone CK3-6H5, Miltenyi Biotec GmbH, Bergisch Gladbach, Germany) with differentiation between proximal TECs by CD-10 (CD10-PE-Vio770, human, clone 97C5, Miltenyi Biotec GmbH, Bergisch Gladbach, Germany), and distal TECs by EpCAM (CD326 (EpCAM)-APC-Vio770, human, clone HEA-125, Miltenyi Biotec GmbH, Bergisch Gladbach, Germany) (22). Cells will be incubated with antibody mixtures for 15 minutes at 4°C in the dark and gated according to positive and isotype-negative controls. For dead cell identification, propidium iodide (Miltenyi Biotec GmbH, Bergisch Gladbach, Germany) will be used to gate out non-viable cells. According to standard protocols, cells will be analyzed with the FACS Canto II machine of the cell sorting unit at the Department for Hematology and Medical Oncology of the UMG.

### Residual material of renal biopsies

During clinical ICI treatment, the indication for renal biopsies in cases of kidney injury depends on the responsible nephrologist. Residual material of renal biopsies can be utilized for additional diagnostic procedures if ICI-related nephrotoxicity is suspected.

### Immunofluorescence

For immunofluorescent stainings, primary antibodies against PD-L1 (ab205921, Abcam, Cambridge, UK) and Alexa Fluor 488 (Invitrogen, Carlsbad, CA) secondary antibodies will be used, nuclear staining will be performed using 4’,6-diamidino-2-phenylindole (Vector Laboratories).

### Immunohistochemistry

Formalin-fixed, paraffin-embedded kidney sections will be deparaffinized in xylene and rehydrated in ethanol containing distilled water. Tissue sections will be stained using antibodies against PD-L1 (ab205921, Abcam, Cambridge, UK), and labeling is performed using Novolink™ Polymer Detection System (Leica Biosystems, Wetzlar, Germany) according to the manufacturer’s protocol. Nuclear counterstain will be performed using Mayer’s Hematoxylin Solution (Sigma, St. Louis, USA).

### Sample size calculation

Due to the explorative design of the current study evaluating potential parameters that affect the risk of irAEs, the sample size calculation is not hypothesis-driven. Based on the feasibility to enroll, analyze, and follow up, a total number of 200 patients was chosen. The aim of the current study is the hazard ratio assessment of different influencing factors on irAEs related to the kidneys.

### Statistical methods

To assess putative correlations between compiled data, regression methods (Cox regression, logistic regression, Poisson regression) will be performed. Moreover, time-to-event analyses and Kaplan-Meier survival estimates will be conducted. Variables will be tested for normal distribution using the Shapiro-Wilk test. Non-normally distributed continuous variables will be expressed as the median and interquartile range (IQR), and categorical variables as frequency and percentage. Statistical comparisons are not formally powered or prespecified. The Mann-Whitney U-test will be used for group comparisons to determine differences between median values. Spearman correlations will be visualized by heatmaps reflecting mean values of Spearman’s ρ. If not otherwise, specified all calculations are performed with GraphPad Prism (Version 9.5.0 for macOS, GraphPad Software, San Diego, California USA, “www.graphpad.com”) or STATA (STATA/MP version 16.1 for Windows, Stata Corp LLC, College Station, TX, USA). Due to the non-interventional, observational, and prospective study design, hypothesis testing will not be conducted.

## Discussion

Kidney affection occurring as an irAE during immunotherapy is a severe and potentially life-threatening complication endangering treatment success, hence the overall survival of cancer patients. Clinically apparent renal involvement is estimated to occur with an incidence of 3%. In contrast, previous investigations of biopsy-proven ICI-related nephrotoxicity reported a much higher frequency of up to 29% of cases implying that conventional diagnostic methods only detect advanced and pronounced kidney injury and are unable to detect early and milder forms of renal involvement (8–16). Renal biopsy is the gold standard for the etiological assignment of kidney affection but carries an increased risk of bleeding complications in the cancer-patient population. Moreover, repeated renal biopsies during a complete treatment course iteratively subject patients to the risk of hemorrhagic events. Despite discontinuing ICI treatment and steroid therapy, the kidneys stay endangered to relapse. In case of renal relapses due to cancer-treatment continuation, the clinical course may even be more fulminant, requiring kidney replacement therapy (KRT) (2). Thus, new tools for biomonitoring in clinical routine will be helpful for the early detection of complications.

Our study aims to evaluate the urinary detection of PD-L1^+^ kidney cells based on urinary flow cytometry as a non-invasive biomonitoring tool for renal complications in cancer patients treated with immunotherapy. The concept of urinary flow cytometry-based detection of renal and immune cells reflecting so-called “biosignatures” was first described for renal allograft pathology (22, 23). Adapted from receiver operating characteristics-(ROC)-curve analyses, an assignment of urinary detected cellular components and renal allograft rejection, including T-cell mediated rejection (TCMR), was enabled (22). The conceptual extension of these findings to other T-cell-mediated renal pathologies, for example, ICI-related nephrotoxicity, seems attractive. Moreover, we previously reported intrarenal PD-L1-positivity as a potential indicator of ongoing kidney damage, showing that renal irAEs correlate with detected PD-L1^+^ kidney cells in the urine (20, 21). PD-L1 upregulation was also observed in other renal pathologies implying PD-L1 functions as a response signal to injuries within the kidneys (20).

Therefore, it is tempting to speculate that the non-invasive urinary detection of PD-L1^+^ kidney cells may enable identifying patients at risk for developing nephrotoxicity related to ICI therapy by urine monitoring.

In summary, in light of the increased use of ICI treatments, we expect a sizable number of renal complications. Therefore, cost-efficient and easily performable diagnostic tools for non-invasive biomonitoring have become more critical to improving renal and overall survival in cancer patients receiving immunotherapy.

## Ethics statement

All enrolled patients will provide written consent. The ethics committee of the University Medical Center Göttingen, Germany, approved the study protocol (Protocol Number 1/10/21). The trial is registered at https://www.drks.de (DRKS-ID: DRKS00030999).

## Author contributions

BT conceived the study and edited the manuscript. EB established protocols and wrote the first draft. PK reviewed and edited the draft and is involved in patient care. AS, K-MT, and TO are directly involved in the treatment of cancer patients. All authors contributed to the article and approved the submitted version.
